# Swine Dysentery: Aetiology, Pathogenicity, Determinants of Transmission and the Fight against the Disease

**DOI:** 10.3390/ijerph10051927

**Published:** 2013-05-10

**Authors:** Avelino Alvarez-Ordóñez, Francisco Javier Martínez-Lobo, Héctor Arguello, Ana Carvajal, Pedro Rubio

**Affiliations:** Infectious Diseases and Epidemiology Unit, University of León, León 24071, Spain; E-Mails: fmarl@unileon.es (F.J.M.-L.); hector.arguello@unileon.es (H.A.); ana.carvajal@unileon.es (A.C.); p.rubio@unileon.es (P.R.)

**Keywords:** swine dysentery, *Brachyspira hyodysenteriae*, transmission, control

## Abstract

Swine Dysentery (SD) is a severe mucohaemorhagic enteric disease of pigs caused by *Brachyspira hyodysenteriae,* which has a large impact on pig production and causes important losses due to mortality and sub-optimal performance. Although *B. hyodysenteriae* has been traditionally considered a pathogen mainly transmitted by direct contact, through the introduction of subclinically infected animals into a previously uninfected herd, recent findings position *B. hyodysenteriae* as a potential threat for indirect transmission between farms. This article summarizes the knowledge available on the etiological agent of SD and its virulence traits, and reviews the determinants of SD transmission. The between-herds and within-herd transmission routes are addressed. The factors affecting disease transmission are thoroughly discussed, *i.e.*, environmental survival of the pathogen, husbandry factors (production system, production stage, farm management), role of vectors, diet influence and interaction of the microorganism with gut microbiota. Finally, prophylactic and therapeutic approaches to fight against the disease are briefly described.

## 1. Introduction

Swine Dysentery (SD) is a severe mucohaemorhagic enteric disease of pigs which has a large impact on pig production, with important losses caused by mortality and sub-optimal performance with reduced feed conversion and gain weight indexes [1]. SD was first described in 1921 [2], but the aetiology was not determined until the seventies, when *Brachyspira hyodysenteriae* was confirmed as the causative agent [3,4]. SD primarily affects pigs during the growth and finishing periods, and clinical signs, which range from mild, mucous diarrhoea with unaltered general condition to severe haemorhagic diarrhoea with a mortality rate of 50–90% [5], seem to occur in a cyclic manner at 3 to 4 weeks intervals, with these recurring symptoms often appearing only after removal of therapeutic antibiotics [6]. 

SD is a widely distributed disease around the World, although studies regarding epidemiology are scarce and the reported prevalence significantly varies among them. Thus, *B. hyodysenteriae* reported prevalence ranges from 0% to near 40% [6,7,8,9,10]. Variations in prevalence can be due to the use of different diagnostic methods or to differences among countries in housing, management, feeding regimes, *etc.* [11,12]. Moreover, whereas in many countries the prevalence may be concealed by the use of antimicrobials as feed additives, in others the ban of antibiotics as growth promoters may have resulted in an increase in SD prevalence [13,14]. 

It is important to note that recent reports have associated clinical dysentery with infection by strongly beta-hemolytic *Brachyspira* spp. that are not confirmed as *B. hyodysenteriae* by PCR and gene sequencing [15,16]. This includes the recently described species *Brachyspira hampsonii* [17]. Interestingly, reproduction of mucohaemorhagic diarrhoea and colitis indistinguishable from SD has been achieved through experimental inoculation with a *B. hampsonii* strain [18]. 

*B. hyodysenteriae* has been traditionally considered a pathogen mainly transmitted by direct contact, through the introduction of subclinically infected animals into a previously uninfected herd [19]. However, recent findings position *B. hyodysenteriae* as a potential threat for indirect transmission between farms, *i.e.*, it can survive for large periods of time in pig faeces, and it has been found in feral pigs, laying chickens, mallards, rheas, seagulls, rodents, dogs, flies and other insects, among others [20,21,22,23,24,25,26,27].

This article summarizes the knowledge available on *B. hyodysenteriae*, the most well-characterized SD agent, including its virulence traits, and reviews the determinants of SD transmission. The between-herds and within-herd transmission routes are addressed. The factors affecting disease transmission are thoroughly discussed, * i.e.*, environmental survival of the pathogen, husbandry factors (production system, production stage, farm management), role of vectors, diet influence and interaction of the microorganism with gut microbiota. Finally, prophylactic and therapeutic approaches to fight against the disease are briefly described.

## 2. The Etiological Agent: General Considerations and Virulence Traits; Lessons from the Genome

*B. hyodysenteriae* is a Gram negative, motile, helically coiled (spiral-shaped), anaerobic bacterium which belongs to the *Brachyspiraceae* Family (*Phylum Spirochaetes*) [28]. *B. hyodysenteriae* is associated with mucus in the lumen and crypts of the porcine caecum and colon, where it causes damage to enterocytes. The lack of genetic tools, and the difficulties involved in its genetic manipulation have hindered the identification of virulence factors and metabolic traits allowing the microorganism to successfully colonize the porcine intestinal tract [29]. However, the first representative genome of a *B. hyodysenteriae* strain (*B. hyodysenteriae* strain WA1), determined in 2009 by Bellgard and colleagues, has shed light on the main adaptations of the species to its lifestyle in the porcine large intestine [30]. In that study, *B. hyodysenteriae* was shown to differ from all the other spirochaetes, including *Leptospira*, *Borrelia* and *Treponema*, in signal transduction and in amino acid transport and metabolism systems. A relative paucity of signal transduction mechanisms relative to the genome size, which probably reflects the relatively narrow ecological niche occupied by the microorganism in the porcine large intestine, was observed. On the other hand, the proportion of genes involved in amino acid transport and metabolism was relatively high, and this probably reflects the adaptation to the environment of the intestinal tract, where proteins from host cells and dietary ingredients are abundant. It was also noteworthy the high proportion of putative protein-coding sequences (CDS) showing high similarity to proteins from the genus *Escherichia* and *Clostridium*. It is likely that these genes were involved in horizontal gene transfer events involving *B. hyodysenteriae* and one or more *Clostridium* and *Escherichia* species. Since they inhabit the same environment in the large intestine, opportunities for gene exchange favouring their survival in this niche are abundant. Several CDS predicted as putative virulence factors were identified. These included proteases involved in virulence via the destruction of host tissues, and ankyrin proteins, known to bind to the host chromatin playing a critical role in the interaction with the host cells. Moreover, seven potential hemolysin production genes, ten flagella-associated genes that can form part of a type III secretory system, at least 84 putative genes associated with chemotaxis and motility, and the key genes necessary for lipooligosaccharide biosynthesis were identified and proposed as virulence factors. In agreement, other studies had previously highlighted the role played by hemolysins, flagella, the lipooligosaccharide and bacterial chemotaxis and motility in SD pathogenesis [31,32,33,34]. Other authors have also identified various virulence life-style factors (e.g., outer membrane proteins, NADH oxidase, proteins of iron metabolism) with a predicted role in *B. hyodysenteriae* pathogenicity [35,36]. The presence of a 35,940 bp circular plasmid in *B. hyodysenteriae* strain WA1 was also confirmed by Bellgard and co-workers [30]. Interestingly, a recent study by La and colleagues [37] has found evidence that this plasmid contributes to *B. hyodysenteriae* virulence. These authors have shown that the WA1 plasmid contains genes encoding enzymes forming part of the rhamnose biosynthesis pathway (*rfb* genes) that are predicted to function in incorporation of rhamnose in the O-antigen backbone of the cell wall lipooligosaccharide. In addition, other glycosyltransferases were shown to be encoded by the plasmid, and these may be involved in incorporating other sugars into the lipooligosaccharide.

## 3. Environmental Determinants of SD Transmission

**Figure 1:**
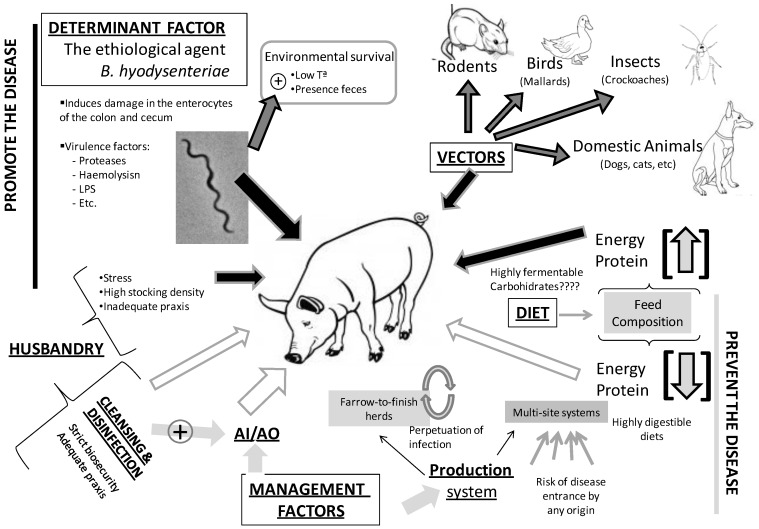
Factors influencing the establishment and transmission of Swine Dysentery (SD). The determinant factor of the disease is the pathogen *Brachyspira hyodisenteriae* whose persistence in the environment is enhanced by low temperatures and the presence of faeces. Factors which promote the transmission, establishment and persistence of the disease are the presence of vectors, diets with high energy and proteins, and bad husbandry practices; the role of highly fermentable diets is unclear. In contrast, all-in/all-out (AI/AO) management together with cleaning and disinfection, strict biosecurity and highly digestible diets are preventive factors to the establishment of SD. Finally, the production system is a decisive factor to a great extent in the control and prevention of the disease. Farrow-to-finish herds have lower risk of SD entrance but in contrast the persistence of the pathogen is higher than in multi-site production systems.

Although SD is a multifactorial disease which pathogenesis is complex and poorly understood, several factors have been associated with the occurrence of the condition. Thus, the outcome of infection by *B. hyodysenteriae* might be influenced by age [38], stress [39], acid secretion in the stomach [40], differences in the dose of the infectious agent, diet and the virulence of *B. hyodysenteriae* strains [41,42]. The following sections of the review emphasize the main environmental factors determining disease establishment and transmission, *i.e.*, environmental survival of the pathogen, husbandry factors, role of vectors, relevance of pig’s diet, and interaction of the microorganism with gut microbiota (Figure 1).

### 3.1. Environmental Survival

Although *B. hyodysenteriae* is an anaerobic pathogen, it is able to survive in the environment of the farm for considerable periods of time, depending on the presence of organic matter and the environmental temperature [22,43,44]. *B. hyodysenteriae* is relatively resistant in the environment of a pig house. It can survive in soil held at 10 °C during 10 days. In the presence of faeces, the survival time is increased to 78 days, and even can reach 112 days in pure pig faeces [22]. Also Chia and Taylor [43] showed that *B. hyodysenteriae* was able to survive for 48 days in dysenteric faeces held between 0 and 10 °C, although it only survived for 7 days at 25 °C, and for less than 24 h at 37 °C. Compared to other spirochaetes, its environmental survival capability is shorter; for instance, *B. pilosicoli*, the agent of the porcine intestinal spirochaetosis, survived for 119 days in pure soil, and 210 days both in soil with 10% pig faeces and in pure pig faeces [22].

### 3.2. Biosecurity and Husbandry Factors

Apart from the etiological agent, other factors also play an important role in the success of the establishment or persistence of the disease. This section of the review deals with handling and biosecurity aspects of SD.

The production system is a decisive factor to a great extent in the control and prevention of the disease. In farrow-to-finish herds (including farrow-to-weaners and farrow-to-growers piggeries), the pathogen can persist in endemic infected sows, which have overcome the infection and developed protective immunity but still shed the pathogen in their faeces. The proximity of facilities and continuous flow of animals in this sort of production system will facilitate the transmission of infection to non-infected animals. On an endemically infected farrow-to-finish piggery transmission of infection to susceptible pigs occurs primarily by contact with faecal material that originates from clinically infected pigs or from asymptomatic carriers colonized by the spirochaete [45]. Depending on the herd immune status and the measures taken to control the disease (based on antimicrobial treatments and vaccination [46,47]), animals will be more or less severely affected and the disease will affect principally pigs at the growing or finishing period when the medication used to control respiratory infections is removed, favouring the expression of SD. In contrast, it is easier to halt the transmission of the pathogen in animals reared in integration systems in which each part of the production is physically independent from the other [48]. However, in integration systems at the growing and finishing stages pigs from different origins are mingled. In consequence these stages constitute a risk if pigs from farms with different status to SD (infected or non-infected) are mixed. Not only productive parameters but also the health status must therefore be considered in the acquisition of pigs. The replacement of breeders with others from the same source each year was shown to be protective against the appearance of the disease [49].

The introduction of all-in/all-out procedures (AIAO) facilitates the disruption of infection transmission among production stages and from consecutive reared batches. The establishment of AIAO requires the cleaning and disinfection of the accommodation together with a period of time during which the barn is empty before it is refilled. Several studies have reported the benefits of using AIAO in SD control. For instance, an increase in the use of antimicrobials to battle SD was registered after the banning of growth promoters in Sweden [50]. However, the introduction of AIAO in Swedish pig production, the closure of many small production units and the increase in size of the remaining herds reversed this situation. Also AIAO management was shown to reduce the odds of being PCR-positive to *Brachyspira* spp. by 4-fold in a study on spirochaetes in poultry [51]. The key in AIAO procedures is the application of efficient protocols of cleaning and disinfection. Considering the susceptibility of *Brachyspira* spp. to the most commonly used disinfectants [43,52,53], the proper application of disinfectants should be effective in removing the environmental spirochaetes present in the herd. However, apart from a thorough disinfection of the pens and corridors, the disinfection programme should also be applied to tools and equipment which could be in contact with faeces and therefore harbour the pathogen. Special attention should be paid to the pit, where *B. hyodysenteriae* can survive for long periods of time. Therefore, manure handling systems must be switched to clean formats [52] by adequate draining, drying and lime applications of manure pits, ponds, *etc.* Effective protocols used 0.15 kg/L of hydrated calcium oxide in slurry channels [54] and calcium oxide 2 kg/m^2^ poured into the canals [55].

Biosecurity aspects are also important for the prevention of disease transmission. These include general aspects, such as the presence of double fence to prevent the entrance of wild animals, and the inclusion of footbaths at the gates of the farm (vehicles) and at the entrance of the barns (caretakers and visitors). Farms should be designed to facilitate feed distribution and cadaver collection preventing the entrance of vehicles which can disperse pathogens from infected farms. Similarly, the presence of a lockable facility for changing clothes which includes farm clothes and boots for caretakers and also visitors is really recommended. In addition, newly acquired pigs should be kept in quarantine facilities for at least three weeks. This process is strongly recommended since clinical signs usually appear in subclinically infected animals as a result of transportation [52]. In the risk factors study performed by Robertson and colleagues [49] some of the aspects mentioned here, double fence, feed transport or visits to farm, were linked to the prevention or presence of SD at the farm.

Finally, certain husbandry factors associated with pig handling could be a potential trigger or enhancer of SD. Asymptomatic pigs may develop diarrhoea following stressful management procedures, such as transferring to new pens, mixture of animals from different origin, weighting or changes in feed [5]. At the same time, adequate stocking densities and temperatures are also factors that have to be considered.

### 3.3. Vectors

The bacterial genus *Brachyspira* consists of intestinal spirochaetes with the capability of colonizing a broad spectrum of hosts. Potential reservoirs of infection on a piggery include feral and other animals [19].

Wild rodents are potential vectors of *Brachyspira* spp. A number of studies have shown that both the brown rat (*Rattus norvegicus)* and the house mouse (*Mus musculus)* are susceptible to *B. hyodysenteriae* infection and have demonstrated the potential transmissibility of the pathogen from mice to pigs [56,57]. Furthermore, typing studies by PFGE have linked strains detected in infected pigs with those isolated from mice and rats from the same farm [58,59,60].

Other important reservoirs are birds. A number of studies have been focused on the isolation of *B. hyodysenteriae* and other spirochaetes from birds with the aim of determining if they constitute a source of infection for production animals and humans [21,23,61,62,63,64,65,66]. Taken together, their results strongly support the conclusion that intestinal spirochaetes are commonly found in the wild-living water-bird species analysed (principally mallards). It is thought that they could play an important role in the transmission of the disease between neighbouring farms and also in the dispersion of the pathogen in their migrations, when the excretion of spirochaetes in faeces is quite frequent [23]. Despite the fact that *B. hyodysenteriae* is frequently isolated from birds the clinical significance of the bacteria in non-porcine species remains somewhat unclear [67,68,69]. Colonization most likely does not cause clinical disease signs in mallards and some degree of host adaptation occurs according to Jansson and colleagues [65]. It is worth mentioning that experimental challenges carried out with *B. hyodysenteriae* isolates from birds have failed to infect pigs [23,70].

Some insect species could carry important enteric disease agents with implications for on-farm spread and maintenance, affecting biosecurity and eradication protocols on pig farms. Insect vectors seem to harbour *Brachyspira* spp. and constitute a reservoir and source of infection for pigs [71]. *B. hyodysenteriae* has been isolated from cockroaches and flies [26]. In the study by Blunt and McOrist [72] five of 14 intestine samples from cockroaches (*Blatta orientalis)* present in SD infected farms were confirmed positive for *B. hyodysenteriae*. Moreover, experimental infection of these cockroaches showed that they remained positive for at least three days after inoculation.

Wild boars may also be a potential source of infection. Philips and colleagues [27] could isolate spirochaetes from feral pigs. In contrast, neither *B. hyodysenteriae* nor any other intestinal spirochaetes were detected in wild boar samples collected in Sweden [73]. Apart from feral animals, domestic animals present in the farms, principally dogs, can be a reservoir of *Brachyspira* spp. as it has been asserted by several authors [74].

Despite the fact that SD is a host specific disease caused by *B. hyodysenteriae* in swine, the information provided in this section demonstrates the importance of other animals, including birds, as potential reservoirs and source of infection for susceptible pigs. These vectors must be taken into consideration when control and, above all, eradication programmes are going to be put under way.

### 3.4. The Role of Diet and Intestinal Microbiota

The pig’s diet has been proposed as one of the most important factors that can influence on spirochaete colonization and on the occurrence of mucohaemorhagic diarrhoea. Particularly, feed composition and abrupt dietary changes have been associated with an increase in the incidence of SD. 

The influence of diet composition on the appearance of SD might be mainly related to the digestibility of their ingredients, which, in turn may have an effect on the composition and equilibrium of the large intestinal microbiota [75,76]. The composition of the microbiota is relevant because *B. hyodysenteriae* executes its pathogenic action in association with other anaerobic members of the large intestinal microbiota to induce extensive inflammation and necrosis of the epithelial surface of the caecum and colon [5]. In addition, changes in the colonic microbiota could either enhance or inhibit the colonization by *B. hyodysenteriae*. The inhibition could be direct or indirect, by inhibition of any of the synergistic bacteria that have been reported to facilitate colonization of this spirochaete [77]. These effects on spirochaete colonization might be reflected in a severe SD after exposure to *B. hyodysenteriae* or in a complete prevention of, or at least a decrease in, the clinical signs of the disease. However, the precise mechanisms by which the diet’s composition predispose to or protect against SD are not fully understood. 

The addition of soybean meal to pig´s diet seems to be a very important factor on the appearance of SD. Thus, pigs experimentally fed large quantities of soya showed clinical signs of dysentery [68]. Moreover, it is known that the addition of a high percentage of soybean meal to meal formulation is associated with both small intestine [78] and large intestine diarrhoea [79]. Although the exact mechanism that predisposes to SD is not yet understood, it is likely that the increase in the protein:carbohydrate ratio in the hindgut associated with a high percentage of soya in the meal plays a role through alterations in the colonic microbiota. 

On the contrary, highly digestible diets reduce the fermentative activity in the large intestine. This fact might contribute to inhibit the colonization by *B. hyodysenteriae* and consequently to prevent the onset of SD. It has been suggested that clinical protection in pigs fed highly digestible diets is due to a reduced fermentation in the large intestine compared to pigs that developed SD [80]. However, protection against SD by feeding pigs with highly digestible diets has not always been achieved [81,82]. Conversely, protection against SD can be achieved with diets supplemented with highly fermentable carbohydrates [83], which produce the opposite effect than highly digestible diets in the hindgut. However, the above mentioned formulation was based on dried chicory roots and sweet lupins, which has allowed speculating with the possibility that the protective effect was due to the presence of inulin in dried chicory roots [84]. Fermentation of inulin by the indigenous microbiota results in the production of volatile fatty acids (VFA) and gases [85], which causes a reduction in luminal pH values in the caecum, upper colon and lower colon. This decrease in luminal pH values might prevent colonization by *B. hyodysentariae* [86]. An alternative mechanism of action would be the regulation of metabolic activity by inulin supplementation, decreasing the protein:carbohydrate ratio in the hindgut and, consequently, the protein fermentation in the hindgut. This fact causes an increase in lactate and butyrate-producing bacteria [87,88] and a reduction in proteolytic bacteria, which could be synergistic with *B. hyodysenteriae* in SD pathogenesis. 

Altogether, these results indicate that the reproducibility of protection against SD by modifications of the diet is low, pointing towards a complex effect in which the diet formulation, including raw materials used and their proportion, the microbiota composition and other yet unrevealed factors play a role in the final outcome of infection. Besides, the inclusion of theoretically protective raw materials in the diet might be too expensive to be routinely implemented in pig diet formulations. More studies are required to clarify the precise role of the diet in the development of the disease and to find economically viable formulations to prevent the condition.

## 4. The Fight against SD

### 4.1. Therapy with Antibiotics

Treatment of SD involves the use of antibiotics. Pleuromutilins (tiamulin and valnemulin) have been used for this purpose in the European Union (EU) [89]. Tiamulin and valnemulin are semi-synthetic derivatives of the naturally occurring diterpene antibiotic pleuromutilin which show outstanding activity against anaerobic bacteria and are used exclusively in animals, largely in swine. Also macrolides (tylosin and, more recently, tylvalosin) and the closely related lincomycin (lincosamide) have been commonly included in SD therapeutic strategies [5] (Table 1). However, the emergence of *B. hyodysenteriae* strains with reduced susceptibility to one or more of these antibiotics and the presence of genetically diverse multiresistant isolates has been confirmed in several countries [90,91,92,93,94,95,96,97,98]. This fact complicates treatment and control of SD and should alert veterinary surgeons and pig farmers for the need of a strategic approach to select antibiotics, which must only be used on strict indications following proper field and laboratory diagnosis in order to guarantee their long-term efficiency for SD treatment.

**Table 1:** Main antimicrobials used for the treatment and prevention of swine dysentery (SD).

Drug	Dosage in SD treatment ^a^	Dosage in SD prevention	Point mutations associated to decreased susceptibility ^b^	Wild type MIC cutoff values ^c^	Clinical MIC breakpoint ^d^
Tiamulin	Im: 10 mg/kg bw for 1–3 days	In feed medication: 30–40 ppm	23S rRNA gene position 2058 and 2032	>0.25 µg/mL	>2 µg/mL
	Po: 8 mg/kg bw for 5–7 days in drinking water		L3 ribosomal protein gene 148		
	In feed medication: 100 ppm for 7–10 days				
Valnemulin	In feed medication: 3–4 mg/kg bw for 1–4 weeks	In feed medication: 25 ppm	23S rRNA gene position 2058 and 2032 L3 ribosomal protein gene position 149	>0.125 µg/mL	>5 µg/mL
Tylosin	Im: 10 mg/kg bw for 3–5 days	-	23S rRNA gene position 2058	>16 µg/mL	>32 µg/mL
	Po: 5–10 mg/kg bw in drinking water for 5–7 days				
Tylvalosin	In feed medication: 4.25 mg/kg bw for 10–14 days	In feed medication: 2.125 mg/kg·bw	23S rRNA gene position 2058 and 2059	>1 µg/mL	>32 µg/mL
Lincomycin	Po: 8 mg/kg bw in drinking water for 1 to 10 days	In feed medication: 44 ppm	23S rRNA gene position 2058, 2059 and 2032	>1 µg/mL	>36 µg/mL
	In feed medication: 100 ppm until clinical signs disappear followed by 40 ppm				

kg: kilogram; mg: milligram; µg: microgram; µl: milliliter; bw: body weight; im: intramuscular; ppm: parts per million; po: per os; ^a^ Information is a summary of several commercial products. For more specific information review product labels; ^b^ This information has been obtained from [97,99,100,101]; ^c^ These cut-off values are solely to monitor any change of antibiotic resistance in the *B. hyodysenteriae* population [102]; ^d^ These data are used for interpreting the clinical outcome of treatment based on data of pharmacokinetic, pharmacodynamic and clinical correlations of drugs [102,103,104].

### 4.2. Vaccination

Large efforts have been made in order to develop vaccines to control SD since Joens and co-authors [105] reported that pigs which have recovered from acute SD are protected from disease when subsequently re-exposed to *B. hyodysenteriae*, indicating that the infection can induce a protective immune response. However, attempts to date have met with limited success. Tested vaccines have included whole-cell bacterins [106,107,108,109] and orally administered attenuated strains [34,110,111,112]. Bacterin vaccines may provide some level of protection but they do not provide adequate cross-protective immunity against strains of different serogroups, which would require the use of autogenous or multivalent bacterins. In addition, they are relatively expensive and difficult to produce on a large scale due to the fastidious growth requirements of the spirochaete. On the other hand, attenuated or genetically modified live avirulent vaccines may show reduced colonisation and cause less immune stimulation. An alternative approach may be to generate subunit vaccines that might be delivered by the expression of recombinant *B. hyodysenteriae* proteins on a bacterial delivery vector [113]. Investigations into potential targets for such recombinant vaccines have focused on outer membrane proteins [114,115,116,117], flagelar proteins [118,119] or iron storage proteins [120], although in most occasions recombinant vaccines tested have failed to provide enough protection in pigs.

### 4.3. Dietary Interventions

As described in previous sections of this review article the pigs’ diet is one of the most important factors regulating the microbial balance in the large intestine and can have a strong influence on colonisation by *B. hyodysenteriae* and on the occurrence or severity of clinical signs of SD. This suggests that control of SD may be achieved by manipulations of dietary ingredients or the use of specific dietary additives. Thus, Siba and co-workers [80] demonstrated that pigs fed a highly digestible diet based on cooked white rice and animal protein were fully protected against SD, while pigs fed diets containing lupins and/or wheat displayed clinical signs of SD. Similar results have been obtained with other diets with low contents in soluble non-starch polysaccharides (sNSP) and resistant starch (RS) [121,122], supporting the idea that microbial digestion of fermentable carbohydrates in the large intestine facilitates the occurrence of SD. However, protection against SD was also shown to be achieved through supplementation with highly fermentable carbohydrates (prebiotic diets; [75,83]). 

Prebiotics are non-digestible food components which evade digestion by mammalian enzymes, in the upper regions of the gastrointestinal tract, reach the colon in an intact state and are then metabolized/fermented by beneficial members of the indigenous microbiota [123]. Selective fermentation by such microorganisms may result in a healthier composition of the gut microflora and a lower susceptibility to gastrointestinal infections. Addition of prebiotics to the diet would therefore allow the manipulation of intestinal microbiota with the final aim of improving health and well-being and preventing SD [88]. Accordingly, a high dietary concentration of inulin, whose possibly mechanisms of action have been mentioned above, has been reported to reduce the incidence of SD in pigs experimentally challenged with *B. hyodysenteriae* [86]. Nonetheless, the reduction of the risk of developing SD was only achieved with a diet supplemented with high levels of inulin (80 g/kg) but not with lower levels, which is an expensive meal formulation. Finally, another dietary additive which has been reported to ameliorate SD, through immunomodulation and reduction of colonic inflammation, is conjugated linoleic acid [124].

### 4.4. Probiotics

As previously mentioned, changes in intestinal microbiota associated with the diet are known to inhibit the development of SD [75], which suggests that supplementation of the diet with strains of beneficial bacteria (probiotics) could protect the host from *B. hyodysenteriae* colonisation. Interestingly, recent reports have identified a range of microorganisms with anti-*Brachyspira* potential [125,126,127]. These included lactobacilli (*L. amylovorus*, *L. farciminis*, *L. rhamnosus*, *L. salivarius*), enterococci (*E. faecium*), bacilli (*B. subtilis*), and bifidobacteria (*B. thermophilum*). The antagonist effects observed by these authors are not yet completely characterized and may be due to direct competition (pathogen displacement) or to the production of bacteriocins or non-specific antimicrobial substances, such as short chain fatty acids. Nevertheless, the anti-*Brachyspira* activity of these strains put them forward as promising probiotic feed supplements in pig production. However, challenge trials are needed to evaluate whether these bacteria exert their antagonistic effects on SD *in vivo*.

### 4.5. Natural Antimicrobials

Intense research efforts focused on the search for alternatives to antibiotics for the prevention of animal infectious diseases have been made in the last decade. Special attention has been paid to the antimicrobial activity of diverse plant and food-derived extracts and their components, which have been reported to show great *in vitro* inhibitory effects against major pathogenic bacteria, including various causative agents of infections in pigs [128,129]. However, little is known regarding *B. hyodysenteriae* susceptibility to this sort of antimicrobial agents. Nonetheless, an extract of citric seeds has been previously reported to inhibit *B. hyodysenteriae* [130], and a recent study by our research group has demonstrated the efficacy of a citrus fruit extract against this pathogenic microorganism [131]. These findings suggest that use of natural antimicrobials as feed supplements could be an attractive approach to control SD. Nevertheless, further research in clinical trials under field conditions is necessary to verify their efficacy without detrimental side effects.

## 5. Conclusions and Future Prospects

SD remains an important endemic infectious disease in many pig rearing countries, where control is limited by the lack of effective vaccines and by the emergence of *B. hyodysenteriae* strains with reduced susceptibility to common antibiotics. 

Despite the great impact of SD, much remains still unknown about the virulence traits of its etiological agent and the transmission modes of the disease. The lack of molecular tools for the genetic manipulation of *B. hyodysenteriae* has hindered the identification of *B. hyodysenteriae* virulence factors. The performance of studies predicting the functions of different genes and macromolecules in the “genomics era” will enable the identification of new virulence factors and regulatory systems important for host colonization by *B. hyodysenteriae*. The publication of the first *B. hyodysenteriae* genome in 2009 has inaugurated this new era, and must serve as a starting signal for the design of ambitious molecular studies which will undoubtedly shed light on the *B. hyodysenteriae* conundrum.

SD is believed to be a multifactorial infectious disease with a complex mode of transmission. Thus, whereas *B. hyodysenteriae* has been traditionally considered a pathogen mainly transmitted by direct contact through the introduction of subclinically infected animals into a previously uninfected herd, recent findings (*i.e.*, great survival ability of the pathogen in faeces, presence of *B. hyodysenteriae* and other *Brachyspira* species in wild boars, domestic animals, rodents, birds and insects) suggest the possibility of indirect transmission between farms. In addition, a range of underlying factors, including the production system, the farm management and the diet of pigs, are relevant for the establishment or transmission of the disease and must be considered when designing control strategies.

Control programmes of SD have been traditionally based on the use of antibiotics. However, the ban on the use of antibiotics as feed additives on farm animals and the emergence of *B. hyodysenteriae* strains with reduced susceptibility to one or more of the used antibiotics, has prompted researchers to search for alternatives in the treatment and prevention of SD. Innovative therapeutic strategies may be focused on the identification or development of novel antimicrobial compounds targeted at the inhibition of bacterial virulence targets, which include drugs inhibiting quorum sensing or biofilm formation. Antimicrobial compounds of natural origin (e.g., plant derived antimicrobials) and with activity on various cellular targets also represent an attractive alternative to conventional antibiotics. The acquisition through genomics-driven studies of novel knowledge on the virulence traits of *B. hyodysenteriae* and on the host immune response to the infection by this microorganism will be essential to progress on the development of a vaccine able to provide full protection against the disease. Finally, preventive measures should be also aimed to act on the underlying factors associated with the disease. This would include the control of the pig’s diet, the modulation of the intestinal microbiota through the inclusion of probiotics and prebiotics on animal feed, or the improvement of farm management practices.
